# Spontaneous intramedullary hematoma following COVID‐19 vaccination: A case report

**DOI:** 10.1002/ccr3.6743

**Published:** 2022-12-19

**Authors:** Arman Sourani, Majid Rezvani, Mina Foroughi, Sadegh Baradaran Mahdavi

**Affiliations:** ^1^ Department of Neurosurgery, School of Medicine Isfahan University of Medical Sciences Isfahan Iran; ^2^ Student Research Committee Isfahan University of Medical Sciences Isfahan Iran; ^3^ Department of Physical Medicine and Rehabilitation, School of Medicine, Student Research Committee, Child Growth and Development Research Center, Research Institute for Primordial Prevention of Non‐Communicable Disease Isfahan University of Medical Sciences Isfahan Iran

**Keywords:** COVID‐19, hematomyelia, neurosurgery, SARS‐CoV‐2, spontaneous intramedullary hematoma

## Abstract

A 67‐year‐old female was hospitalized due to right‐sided hemiparesis and neck pain with rapid deterioration to a deep coma. She had received the Sinopharm vaccine 2 days earlier. MRI showed extensive cervicothoracic hematomyelia. She received intensive medical care for 2 months and was discharged. An 18‐month follow‐up showed significant neurological recovery.

## Abstract 2

COVID‐19 vaccines are relatively safe, but serious complications are expected regarding their emergency approval. Hematomyelia, such as other spinal cord lesions, can be expected in the post‐vaccination period, emphasizing higher healthcare providers' awareness.

## 1 INTRODUCTION

Severe acute respiratory syndrome coronavirus 2 (SARS‐CoV‐2) is a virulent subtype of the Coronaviridae family causing COVID‐19 pneumonia. Headache, peripheral neuropathy, intracerebral hemorrhage, stroke, encephalitis, and Guillain–Barré syndrome are the most common nervous system manifestations of SARS‐CoV‐2.^1^, ^2^ Myelitis, neuropathy, spinal cord dysfunction, and hemorrhagic lesions are amongst the reported spinal manifestations of SARS‐CoV‐2.^2^, ^3^, ^4^, ^5^

SARS‐CoV‐2 vaccines are generally safe and effective in preventing COVID‐19 pneumonia in emergency global SARS‐CoV‐2 situation.^6^ Injection site reactions, fever, headache, myalgia, and rash are the most common vaccine reactions.^7^ However, adverse neurological events such as myelitis, Bell's palsy, demyelinating neuropathy, and cerebral venous embolism are reported following COVID‐19 vaccination, but exact neuropathogenesis is still obscure.^8^, ^9^ Spontaneous intramedullary hematoma or hematomyelia is a devastating medical condition that causes serious mortality and morbidity. Tumors, anticoagulants, and vascular lesions are associated with hematomyelia; still, the majority of them are idiopathic.^10^, ^11^ Although the association between COVID‐19 infection and hematomyelia was previously reported, there is no literature on COVID‐19 vaccines and hematomyelia coincidence.^12^

In this case, we report a rare coincidence of spontaneous intramedullary hematoma following the Sinopharm anti‐COVID‐19 vaccine.

## 2 CASE PRESENTATION

A 67‐year‐old female known case of hypothyroidism was admitted to the emergency department due to right‐sided hemiparesis and neck pain in March 2021. She was agitated but fully conscious and cooperative. According to her history, she had received her first Sinopharm vaccine shot 2 days earlier and, after 24 h, started to feel weak and painful on her right side. The pain was radiating from the right side of the neck to her right extremities. The pain quality was burning in nature and accompanied by a tingling sensation in pain distribution areas. The weakness was more severe in her right hand and elbow than in her right knee and ankle. Her vital signs were stable in physical examination, and her Glasgow coma scale (GCS) score was 15 with normal reactive pupils. Incidental right‐sided mild facial paresis was detected. The rest of the cranial examination showed no specific abnormality. Her manual muscle strength testing (MMT) showed right‐sided hemiparesis and hypoesthesia–dysesthesia, right upper extremity force 3/5, and right lower extremity 4/5. She had controlled long‐standing hypothyroidism and was receiving 100 μ grams of Levothyroxine daily. She was not receiving any anticoagulants or anti‐platelets. Her PT, PTT, INR, D‐dimer, and platelet counts were in normal range. Her daughter mentioned she had a benign skin melanoma that was excised 6 years ago and was under regular dermatologist follow‐up. There was no history of autoimmune diseases. The rest of the medical history and laboratory data were negligible.

### 2.1 Workups and treatments

**FIGURE 1:**
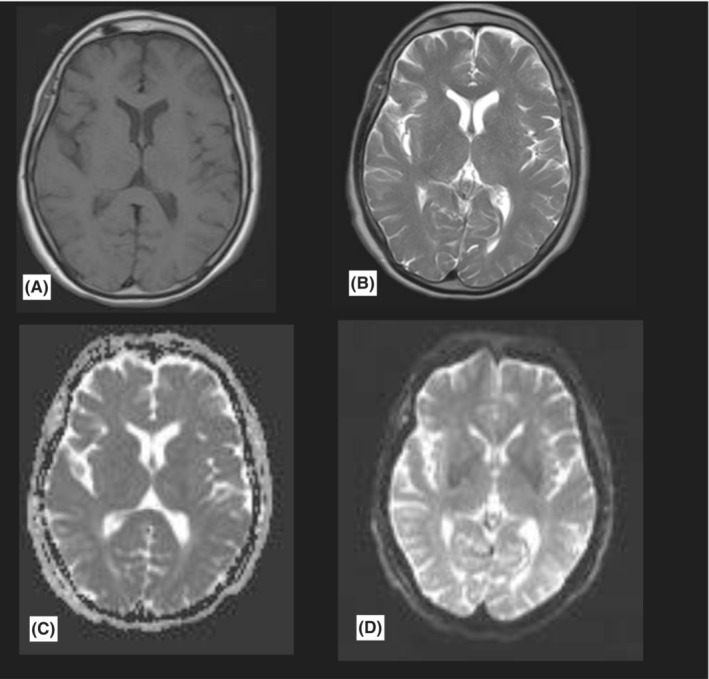
Brain MRI in serial sequences shows no acute intracranial lesions. A = T1, B = T2, C and D = perfusion‐diffusion sequences.

**FIGURE 2:**
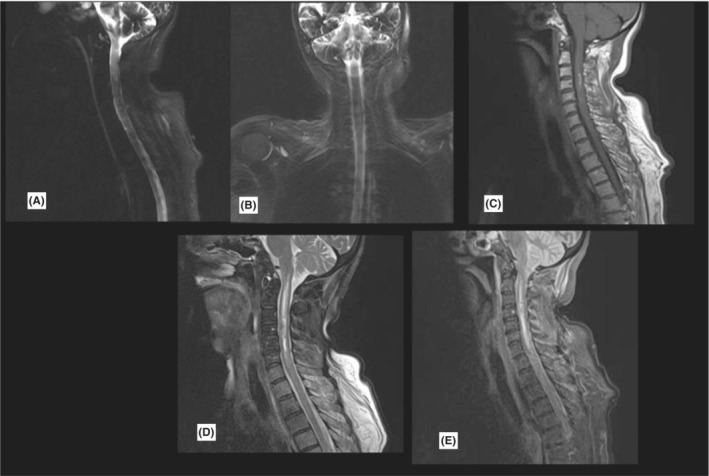
Cervicothoracic MRI demonstrates a hemorrhagic core corresponding to C3–C5 vertebral bodies with extensive cord edema originating from brain stem‐to‐upper thoracic cord myelography images showed no CSF block. A and B = myelograms, C = T1 sagittal view, sequence D = T2, sequence E = STIR image.

Routine blood tests were obtained. According to her presentations, an ongoing stroke or carotid dissection was suspected for her. Unfortunately, 10 min after admission, she experienced a rapid onset of abdominal pain, seizure‐like movements, loss of consciousness, and bradycardia. Rapid endotracheal intubation, hemodynamic support, and 3 min of resuscitation were performed, and she was stabilized. Serial EKG and troponin tests showed no acute cardiologic problems. Brain CT scan showed no acute intracranial process, so she was transferred to the neurological ICU. After further stabilization, a brain MRI was obtained to rule out stroke within an hour. Brain MRI sequences showed no acute stroke or other intracranial lesions (Figure 1). Incidentally, the upper cervical spine seemed edematous compared with the normal spine, so a full spine MRI was taken. There was signal hyperintensity in T1, T2, and short‐tau inversion recovery (STIR) sequences of the spinal cord originating from the brain stem extending to T2 compatible with acute intramedullary hematoma of the cervicothoracic cord. Based on MRI sequences, the hemorrhagic segment corresponded to C3‐C5. There was no cerebrospinal fluid (CSF) blockage in myelograms (Figure 2). Due to a history of previous benign melanoma, we had considered melanoma metastasis to the spinal cord. However, MRI images were not consistent with metastasis but compatible with spontaneous intramedullary hematoma(hematomyelia). Her SARS‐CoV‐2 PCR was negative in serial samples. Her HRCT had no specific pathological point except minor aspiration pneumonia. Due to possible differential diagnoses and the rarity of the disease, multiple medical measurements were taken. Five sessions of plasmapheresis and high‐dose methylprednisolone therapy were used that had no significant clinical effect. She was consulted with the neurosurgery team for possible neurosurgical interventions. Considering her medical status, GCS of 3/15, hemodynamic lability, vasopressor dependency, and the absence of mass effect on the spinal cord, the consultant neurosurgeon recommended conservative treatments over surgical intervention. Over 2 months of ICU care, she gained consciousness, weaned from ventilator assistance, and vasopressors were tapered and discontinued. She was transferred to a general ward and discharged after 2 weeks.

### 2.2 Follow‐up

**FIGURE 3:**
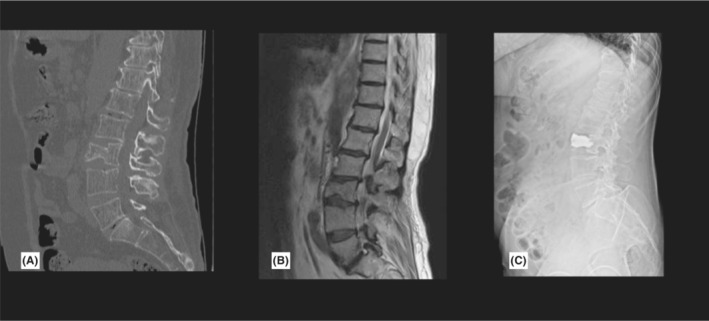
L3 burst fracture with negligible canal compromise in MDCT (A) and MRI (B). Post kyphoplasty x‐ray showed proper vertebral expansion and augmentation(C).

In follow‐up appointments, her modified Rankin Scale (mRS) was 3, and facial paresis was slightly improved. Right‐sided upper extremity MMT was 3/5, and right lower extremity force was 4/5. She was able to walk with walker assistance. Two months later, she took SARS‐CoV‐2 pneumonia and recovered in 2 weeks. After 8 months, she had a ground‐level falling during daily activities that caused a type A3 burst fracture in the L3 vertebral body and underwent cement kyphoplasty (Figure 3). She became independent after 18 months, and her mRS improved to 1.

## 3 DISCUSSION

The proposed mechanism of SARS‐CoV‐2 neuropathogenesis is not established thoroughly. However, endothelial damage in the blood–neuron interface, retrograde axonal transmission, infected leukocyte‐mediated transportation (Trojan horse), cytokine storming, immune complex‐mediated reactions, angiotensin‐converting enzyme‐2 (ACE‐2) mediated virus invasion, and thrombotic microangiopathy are the most accepted theories.^13^, ^14^, ^15^ Myelitis, neuropathy, Miller–Fisher syndrome, Guillain–Barre syndrome, epidural abscess, and spinal cord infarction are the most reported SARS‐CoV‐2 spine lesions.^3^, ^16^, ^17^ Hemorrhagic spine lesions in SARS‐CoV‐2 are exceedingly rare. Lim et al.^18^ reported a cervical spine spontaneous epidural hematoma in a COVID‐19‐infected woman treated conservatively. Zubeyde et al.^5^ reported another epidural hematoma in an older woman positive for SARS‐CoV‐2 virus that underwent laminectomy and hematoma evacuation.

The pathophysiology associated with nervous system manifestations of COVID‐19 vaccines is not clearly understood. It is rational to presume that virus and vaccines share common pathways in immunologic responses. The degree of virus inactivation–reactivation, immune complex‐mediated reactions, cytokine‐mediated systemic response, and vector‐associated complications are suggested.^15^, ^19^ The SARS‐CoV‐2 neuropathogenesis can be attributable to these symptoms since some patients develop COVID‐19 pneumonia in some vaccines. Based on American neurological association (ANA) data, Goss et al.^20^ published a confirmatory article regarding the serious neurological side effects of COVID‐19 vaccines and disclosed possible hazards.

Lu et al.^19^ reviewed the safety and side effects of COVID‐19 vaccines. They discussed vaccine side effects and concluded that vaccines for SARS‐CoV‐2 prevention are generally safe and effective. Immunization stress‐related response triggered by an attenuated virus, vectors, or nucleic acid‐mediated antigenic proteins was introduced as the main step in adverse reactions of the COVID‐19 vaccine.^19^

Finstere and Scorza narrated on Lu's review, disagreed with their safety concepts, and tried to disclose post‐vaccination strokes, neuropathy, myelitis, and acute disseminated encephalomyelitis (ADEM) neuropathogenesis.^21^

Fan et al.^7^ reviewed the existing literature and concluded that mRNA vaccines are associated with lower COVID‐19 infection but with more serious vaccination side effects. Wu et al.^6^ published a review on COVID‐19 vaccination side effects that confirmed Fan's results.

The Sinopharm vaccine is based on inactivated SARS‐CoV‐2 virus administered in multiple doses via intramuscular injection. Kaabi et al. conducted an extensive study on the safety and side effects of the Sinopharm vaccine on approximately 11,000 personnel. They reported 153 SARS‐CoV‐2 PCR‐positive individuals after two doses of vaccination, 92 people with mild COVID‐19 pneumonia, and two cases requiring hospitalization for their post‐vaccination COVID‐19 pneumonia.^22^ Regarding the current literature, there are many controversies regarding neuropathogenesis, safety, and adverse reaction over COVID‐19 vaccines, demanding further reports and investigations.

Spontaneous intramedullary hematoma or hematomyelia is an ominous neurological event. Although predisposing factors such as tumors, vascular malformations, and coagulopathic conditions are associated with hematomyelia, most reports delineate no specific reason.^10^, ^23^, ^24^, ^25^ Sabouri et al.^12^ published the first hematomyelia reported in a 73‐year‐old man with severe COVID‐19 pneumonia. In this report, we presented the first report of hematomyelia after SARS‐CoV‐2 vaccination. Despite the Sabouri et al. report, this patient had no significant past medical record except hypothyroidism and distant history of a small benign melanoma in her hand that was excised 6 years ago, and follow‐up examinations had no malignant transformation or new lesions. On the other hand, neuroaxis MRI sequences ruled out the metastatic or structural lesions in her nervous system. Follow‐up MRI also showed hematoma resolution with focal gliosis/hemosiderin deposition and no apparent underlying lesion. However, the authors would like to emphasize that they only report coincidences of hematomyelia and Sinopharm vaccination logically; this single report neither approves nor denies the possible association between two different entities. The authors would like to encourage clinicians about more reports on this topic for better clinical generalizability.

## AUTHOR CONTRIBUTIONS

**Arman Sourani:** Conceptualization; investigation; project administration; validation; writing – original draft; writing review and editing. **Majid Rezvani:** Investigation; project administration; supervision; validation; writing – review and editing. **Mina Foroughi:** Conceptualization; data curation; supervision; validation; visualization; writing – original draft; writing – review and editing. **Sadegh Baradaran Mahdavi:** Data curation; investigation; methodology; supervision; validation; visualization; writing – original draft; writing – review and editing.

## FUNDING INFORMATION

Not applicable.

## CONFLICT OF INTEREST

The authors declare no conflict of interest.

## ETHICAL APPROVAL

All procedures performed were under the institutional and/or national research committee's ethical standards and the 1964 Helsinki Declaration and its later amendments or comparable ethical standards. This report was supervised and approved by Isfahan University neurosurgery department board members on behalf of the Ethical Committee of Isfahan University of medical sciences.

## CONSENT

Informed consent was obtained from all individual participants included in the Study.

## Data Availability

Data and original images in the current study are available from the corresponding author on reasonable request. Authors can confirm that all relevant data are included in the article and/or its supplementary information files.
